# Crossroads of Care: Navigating Injection Drug Use-Associated Endocarditis

**DOI:** 10.7759/cureus.62490

**Published:** 2024-06-16

**Authors:** Anton Stolear, Maxim Dulgher, Lila Kaminsky, Fabio Ramponi, Gilead Lancaster

**Affiliations:** 1 Cardiology, Yale University/Bridgeport Hospital, Bridgeport, USA; 2 Internal Medicine, Nuvance Health/Norwalk Hospital, Norwalk, USA; 3 Cardiothoracic Surgery, Yale School of Medicine, Bridgeport, USA

**Keywords:** staph aureus bacteremia, septic emboli, substance use disorder, tricuspid valve surgery, injection drug use, infective endocarditis

## Abstract

Infective endocarditis (IE), with its high morbidity and mortality, is a frequent complication of injection drug use (IDU). We present a case highlighting the complexities in the management of IDU-associated IE (IDU-IE) in a 46-year-old male with active IDU who presented with methicillin-susceptible Staphylococcus aureus (MSSA) bacteremia and a large tricuspid valve vegetation. Urgent tricuspid valve surgery was indicated due to the size of the vegetation measuring up to 4 cm, along with recurrent pulmonary septic emboli. The patient underwent an uncomplicated and successful complete vegetectomy, tricuspid valve repair, and completed a 42-day antibiotic course. During the six-week follow-up, he showed complete recovery and maintained successful abstinence from illicit drug use, supported by an addiction medicine specialist.

This case underscores the importance of early recognition, appropriate antibiotic therapy, and individualized surgical intervention in optimizing outcomes. Effective management of IE necessitates a multidisciplinary IE team, including addiction medicine specialists. Addressing the underlying substance use disorder (SUD) is crucial to reducing the risk of recurrent IE.

## Introduction

Infective endocarditis (IE) is a life-threatening condition associated with significant morbidity and mortality. With the escalation of opioid addiction to epidemic proportions in the United States, there has been a corresponding rise in injection drug use (IDU)-IE, posing various challenges including medical, surgical, psychosocial, ethical, and decision-making dilemmas [1]. IDU is a well-recognized predisposing condition of IE, representing one of the minor Modified Duke Criteria for its diagnosis. IE can have devastating consequences for individuals who inject drugs (PWID), particularly as it affects a generally younger and predominantly healthy group of patients [2].

This study aims to illustrate the complexities involved in the management of IDU-IE. Our case emphasizes the critical importance of early diagnosis, appropriate antibiotic therapy, and individualized surgical intervention. It also highlights the need for an IE team - a multidisciplinary approach that includes addiction medicine to address underlying substance use disorder (SUD), ultimately aiming to improve patient outcomes, reduce recurrence rates, and lower healthcare costs.

## Case presentation

We report a case of a 46-year-old male with a history of active SUD on methadone therapy and active intravenous fentanyl use, who presented to our tertiary hospital emergency department with complaints of back pain after a mechanical fall. On physical exam, there was a holosystolic murmur at the left lower sternal border and decreased breath sounds at bilateral bases. There were needle track marks in the bilateral inguinal area and fingernail splinter hemorrhages. There was no significant lower extremity edema. Imaging studies revealed scattered bilateral lung nodules highly suspicious for septic emboli, multifocal pneumonia, and suspected septic arthritis at the L2-L3 facet joint. 

Blood cultures were obtained on admission, and empirical antibiotic therapy with vancomycin, piperacillin, and tazobactam was started. Subsequent septic workup results revealed bacteremia caused by methicillin-susceptible Staphylococcus aureus identified by polymerase chain reaction (PCR), and antibiotic therapy was adjusted to oxacillin based on microbiological susceptibility data under the guidance of the Infectious Disease Service.

**Figure 1:**
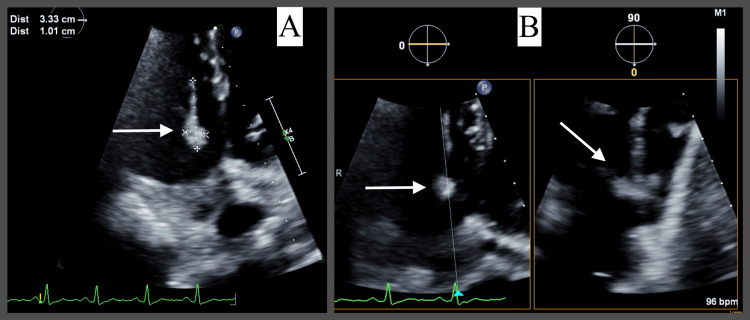
Transthoracic echocardiography (TTE) findings of large, mobile vegetation measuring 3.3 cm x 1 cm (indicated by the white arrows) attached to the tricuspid valve leaflet. A: Apical four-chamber view zoomed on tricuspid valve vegetation. B: Apical four-chamber view with biplane visualization of tricuspid valve vegetation.

Transthoracic echocardiography (TTE) revealed a left ventricular ejection fraction (LVEF) of 57% and normal right ventricular size and function. There was no interatrial shunt detected by color Doppler. A large, mobile mass measuring 3.3 cm x 1 cm was observed attached to the tricuspid valve leaflet, consistent with bacterial vegetation (Figure 1). Quantitative assessment of tricuspid valve regurgitation was challenging due to the presence of vegetation, but it appeared to be mild.

**Figure 2:**
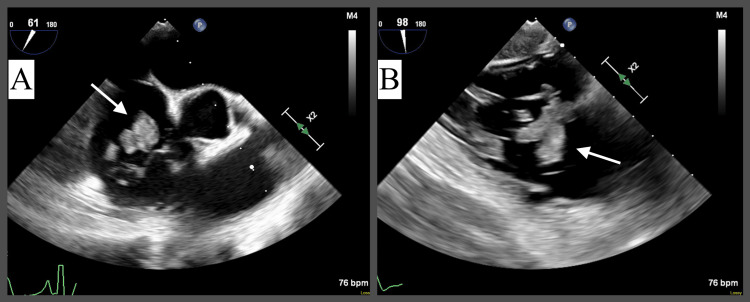
Preoperative transesophageal echocardiogram (TEE) with evidence of large, irregularly shaped, highly mobile vegetation (indicated by the white arrows) on the tricuspid valve leaflet measuring approximately 4 cm x 2 cm. A: Mid-esophageal view at 61° focusing on tricuspid valve vegetation. B: Transgastric view at 98° focusing on tricuspid valve vegetation.

**Figure 3:**
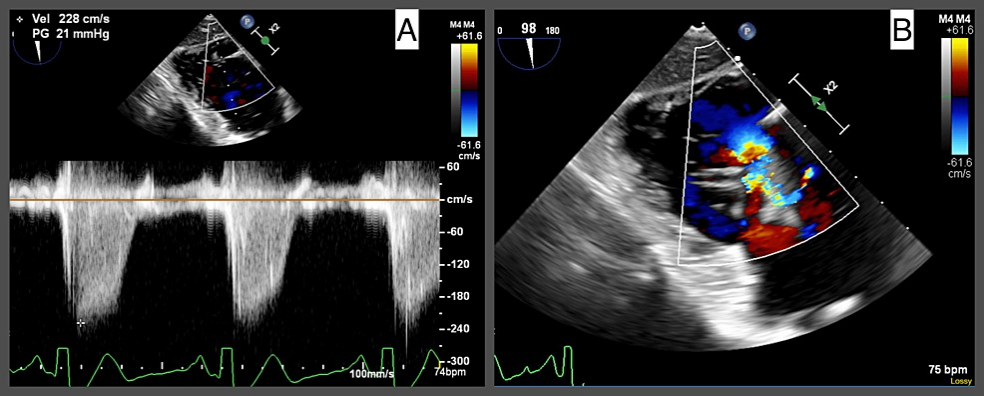
Transesophageal echocardiogram (TEE) evidence of moderate tricuspid regurgitation. Transgastric view. A. Tricuspid regurgitation quantification using continuous wave Doppler velocity. B. Tricuspid regurgitation quantification using color Doppler.

A subsequent transesophageal echocardiogram (TEE) revealed a large, irregularly shaped, highly mobile mass on the tricuspid valve leaflet measuring approximately 4 cm x 2 cm, consistent with vegetation (Figure 2). There was moderate tricuspid regurgitation (Figure 3).

The patient was managed by a multidisciplinary IE team including internal medicine, cardiology, infectious disease, addiction medicine, and cardiothoracic surgery. Despite negative blood cultures on day five, new septic pulmonary emboli were detected on imaging workup. Given the size of the tricuspid valve vegetation and recurrent septic emboli, urgent cardiothoracic surgical intervention was recommended.

**Figure 4:**
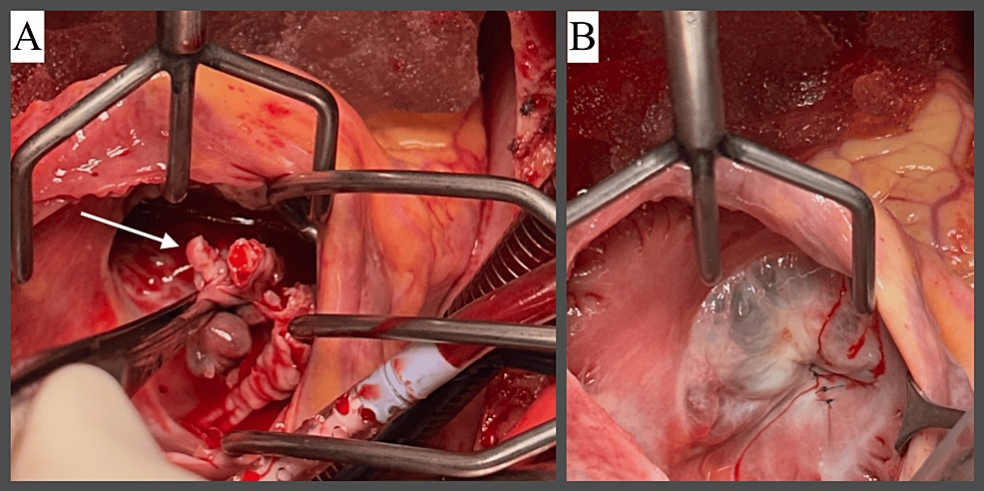
Intraoperative findings. A. Tricuspid valve vegetation (indicated by the white arrow). B. Repaired tricuspid valve with successfully reconstructed septal leaflet.

Uncomplicated tricuspid valve complete vegetectomy and tricuspid valve repair were performed, with successful reconstruction of the septal leaflet (Figure 4). Considering active IDU and the small tricuspid annular size, the decision was made to not proceed with annuloplasty to avoid prosthetic material. 

The postoperative hospital course was uneventful. TTE assessment revealed normal LVEF and mild tricuspid valve regurgitation. The patient was discharged to a skilled nursing facility on postoperative day seven to complete a total of 42 days of antibiotic therapy. Methadone therapy was maintained throughout the hospital stay and following discharge.

At the six-week follow-up, he had achieved complete recovery and sustained successful abstinence from illicit drug use with the assistance of an addiction medicine specialist. 

## Discussion

The incidence of IDU-IE is steadily increasing, carrying a recognized risk that is 100 times greater than that observed in the general population. Caring for these patients is linked to prolonged hospital stays, increased rates of readmission, higher instances of recurrent IE, increased mortality, and higher healthcare costs [3]. Hospitalizations for IE management have largely been driven by a surge in IDU-IE cases. From 2000 to 2013, a nationally representative sample of US hospitalizations revealed a 38% overall increase in IE hospitalizations. In contrast, hospitalizations for IDU-IE rose by 238% [4].

The primary microorganism responsible for right-sided IE is Staphylococcus aureus, which accounts for most cases. In right-sided IE, the tricuspid valve is significantly more often infected than the pulmonary valve [5].

The management of IDU-IE parallels that of non-IDU-IE, involving medical or combined medical-surgical therapy. Comprehensive care should address underlying SUD. Decision-making regarding valve surgery for IE, whether drug-related or not, must be individualized [4]. 

The commonly recognized indications for surgical treatment of right-sided IE based on the most recent guideline of the European Society of Cardiology include heart failure secondary to severe tricuspid regurgitation non-responsive to diuretics, involvement of left-sided structures, persistent bacteremia despite appropriate antibiotic therapy, respiratory failure secondary to recurrent pulmonary emboli necessitating ventilatory support, and large residual tricuspid vegetations (>20 mm) after recurrent pulmonary emboli [5]. In a study of 134 adults undergoing surgical treatment of right-sided IE, a combination of large vegetations and septic pulmonary emboli was the most cited indication for intervention in IDU-IE [6]. Variations in the measured size of vegetations on native valves between TTE and TEE, as presented in our case, may be attributed to significant differences in the sensitivity of both tests. In native valve IE, the diagnostic accuracy of TTE relies on vegetation size and underlying valvular disease, demonstrating a sensitivity ranging from 40% to 63%, while TEE exhibits a sensitivity ranging from 90% to 100% [7]. 

Embolic events significantly increase morbidity and mortality. They occur in 20-40% of patients with IE [8]. Early surgical intervention in these circumstances, when compared to traditional medical treatment, has been shown to significantly lower the combined risk of all-cause mortality and embolic events. This strategy is especially beneficial for patients with IE who have large vegetation, as it was presented in our case. An early surgical strategy effectively reduces the likelihood of systemic embolism in these circumstances [9].

The primary surgical approaches for tricuspid valve IE typically involve repair, replacement, and, to a lesser extent, valvectomy. Tricuspid valve repair is commonly preferred over replacement in cases of right-sided IE, although extensive valve damage may impede repair. Notably, tricuspid valve repair has shown better short- and long-term outcomes than replacement, particularly in reducing recurrent infections and requiring subsequent surgeries. In cases where replacement is required, bioprosthetic valves are often preferred. This consideration stems from the complexities and risks associated with lifelong anticoagulation management. Additionally, there is a heightened potential for thromboembolism with mechanical valves, particularly in patients with a history of IDU [5].

Data from the Society of Thoracic Surgeons Adult Cardiac Surgery Database indicates that between July 2011 and June 2018, there were 34,905 valve operations performed for IE, with 33.7% of these operations attributed to IDU-IE. Operations for this indication increased 2.7-fold during the study period [8]. There is no difference in in-hospital and 30-day mortality between patients undergoing valve surgery for IDU-IE and those in the non-IDU-IE patient population [10]. 

Without addressing SUD, PWID exhibit a significantly elevated risk of recurrent IE, primarily due to ongoing IDU. This heightened risk markedly compromises long-term outcomes, underscoring the necessity for integrating comprehensive SUD treatment strategies to improve prognosis and reduce the incidence of recurrent IE in this population. In North America, the one-year mortality rates have been reported to range from 16% to 20% [4]. In a Boston cohort, a mortality rate of 26% was observed at a median follow-up of 306 days, with the median age of death being 41 years. These findings highlight the profound impact of IDU-IE on young individuals [11]. In another study that analyzed US death certificates, it was found that deaths attributed to IDU-IE increased threefold from 1999 to 2016, while overall deaths related to IE rose by 1.5 times [12]. While there is limited data available on long-term postoperative outcomes exceeding five years, one meta-analysis indicated a five-year and 10-year survival rate of 62% and 57%, respectively. The study also indicated that PWID had a 47% higher risk of death and more than double the risk of reoperation compared to non-PWID [13].

Recent scientific progress in addiction medicine shows that involving addiction therapy specialists can notably decrease mortality and morbidity rates in these patients. For individuals admitted with IDU-IE, the inclusion of addiction specialists in the care team plays a crucial role in improving outcomes by providing targeted support and interventions, addressing the underlying SUD, and helping to prevent the recurrence of IE. Integrating addiction treatment into the overall care plan is essential for enhancing patients’ recovery and long-term outcomes [5,8,10]. 

One innovative approach to addressing IDU-IE involves close outpatient follow-up with integrated infectious disease and substance use disorder care teams, which have been associated with improvements in healthcare utilization, management of IDU, and completion of antibiotic treatment for PWID with infections [14]. Another emerging treatment approach for the management of IDU-IE includes oral antibiotic therapy and long-acting glycopeptides, which have extended half-lives allowing for weekly dosing. These options are particularly appealing for individuals requiring parenteral therapy for prolonged durations [4].

Access to treatment for IDU-IE remains one of the primary challenges in management. This barrier is exacerbated by psychological, social, and economic factors, including mental illness, homelessness, and lack of medical coverage. Consequently, addressing these issues remains a significant challenge.

## Conclusions

This case highlights the importance of a multidisciplinary approach in the management of IE, particularly in patients with underlying SUD. Early recognition, timely initiation of antibiotic therapy, and consideration of surgical intervention when indicated, are crucial in optimizing patient outcomes. Treatment must not stop at the resolution of the IE infection but needs to incorporate comprehensive care to address the underlying SUD. Further research is needed to better understand the impact of substance abuse on the pathogenesis and management of IE, novel treatment modalities, predictive factors for outcomes, as well as decreased recurrence of IE, hospital readmissions, strategies to improve access to SUD treatment, outcomes, and cost of care in this population.
